# The mitochondrial protectant SS31 optimized decellularized Wharton's jelly scaffold improves allogeneic chondrocyte implantation-mediated articular cartilage repair

**DOI:** 10.1016/j.jot.2025.03.023

**Published:** 2025-04-15

**Authors:** Chao Wang, Hao Li, Fakai Li, Yongkang Yang, Ziheng Xu, Tianze Gao, Runmeng Li, Ruiyang Zhang, Yuhao Mu, Zheng Guo, Quanyi Guo, Shuyun Liu

**Affiliations:** a Institute of Orthopedics, The First Medical Center, Chinese PLA General Hospital, Beijing Key Lab of Regenerative Medicine in Orthopedics, Key Laboratory of Musculoskeletal Trauma & War Injuries PLA, No.28 Fuxing Road, Haidian District, Beijing, 100853, China; b School of Medicine, Nankai University, Tianjin, 300071, China

**Keywords:** Articular cartilage, Decellularized Wharton jelly, SS31 peptide, ACI, Regenerative medicine

## Abstract

**Background:**

The process of allogeneic chondrocyte implantation entails obtaining donor chondrocytes, culturing them in a medium enriched with growth factors, and then introducing them-either individually or in conjunction with biocompatible scaffolds-into areas of cartilage damage. While promising, this approach is hindered by mitochondrial dysfunction in the implanted chondrocytes.

**Methods:**

This research introduced an innovative approach by creating a new type of scaffold derived from Decellularized Umbilical Cord Wharton's Jelly (DUCWJ) extracted from human umbilical cords. The scaffold was manufactured using procedures involving decellularization and lyophilization. The resulting scaffold demonstrated superior characteristics, including high porosity, hydrophilic properties, and excellent biocompatibility. To enhance its function, SS31 peptides, known for their mitochondrial-protective properties, were chemically bonded to the scaffold surface, creating an SS31@DUCWJ system. This system aims to protect chondrocytes and regulate the mitochondrial respiratory chain (MRC), thereby improving cartilage repair mediated by allogeneic chondrocyte implantation.

**Results:**

In vitro studies have shown that SS31 effectively attenuates metabolic dysfunction, extracellular matrix degradation, oxidative stress, inflammation, and mitochondrial damage induced by serial cell passages. Complementary in vivo experiments showed that the SS31@DUCWJ scaffold promoted regeneration of healthy articular cartilage in femoral condylar defects in rabbits.

**Conclusions:**

This SS31-modified porous decellularized scaffold represents an innovative biomaterial with anti-inflammatory properties and targeted mitochondrial regulation. It offers a promising new approach for treating articular cartilage injuries.

**The translational potential of this article:**

Our study was the first to successfully load the mitochondrial protectant SS31 onto a DUCWJ hydrogel scaffold for localized drug delivery. This method is highly efficacious in repairing cartilage defects and offers a promising new avenue for the treatment of such conditions.

## Graphical abstract

**Figure:**
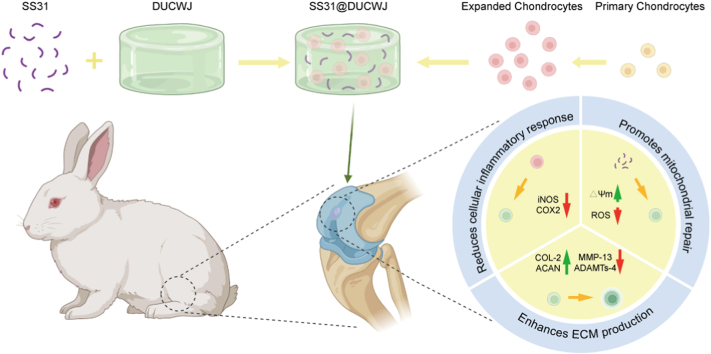

## 1 Introduction

Hyaline articular cartilage consists of chondron clusters embedded within a dense and highly organized extracellular matrix composed primarily of type II collagen, proteoglycans (e.g., aggrecan), and glycosaminoglycans (e.g., hyaluronan) [1]. This unique tissue lines joint surfaces, providing both the strength needed for weight-bearing and the smoothness required for frictionless movement. However, its lack of blood vessels, nerves, and lymphatics, along with the absence of an intrinsic stem cell niche, articular cartilage has a very limited self-healing capability. Consequently, injuries from trauma—common in young individuals, particularly athletes—or age-related wear and tear often fail to repair naturally, eventually leading to osteoarthritis, a debilitating condition affecting millions worldwide [2].

To address this challenge, researchers have developed various techniques for repairing cartilage defects. Procedures like Pridie drilling and microfracture, which create access to bone marrow stem cells, are cost-effective and straightforward but result in fibrocartilage formation. This tissue, unlike native hyaline cartilage, lacks the biochemical and biomechanical properties required for durable clinical outcomes [3]. Regenerative techniques like mosaicplasty and osteochondral autografts provide improved outcomes but have notable downsides, such as the risk of injury at the donor site and potential graft failure due to inadequate integration with the adjacent tissue [4]. In recent decades, the rise of cellular therapies has brought renewed attention to autologous chondrocyte transplantation. While promising, this method requires two surgeries—one to harvest chondrocytes from the patient and another to implant the cultured cells—and often fails to generate sufficient cell numbers for treating larger defects [5]. Against this backdrop, allogeneic chondrocytes have emerged as a viable alternative for cartilage regeneration, offering the potential to overcome these limitations and improve outcomes.

In allogeneic chondrocyte implantation (ACI), chondrocytes are extracted from the articular cartilage of deceased donors, cultured with growth factors in monolayer systems, and implanted into cartilage defects with or without the aid of biocompatible scaffolds. This technique offers several advantages, including the resemblance of the newly formed cartilage to native hyaline cartilage, the absence of ethical concerns associated with harvesting, no need for multiple surgeries, reduced patient morbidity, and access to a large chondrocyte supply. These attributes position ACI as a promising approach for cartilage repair [6,7]. However, despite its potential, ACI faces significant challenges in both laboratory and clinical settings. A critical limitation is the inability of transplanted chondrocytes to integrate effectively with surrounding tissues when implanted alone [8]. This shortcoming highlights the need for a suitable biomaterial scaffold to support chondrocyte culture and transplantation. An ideal scaffold remains difficult to achieve, as it must fulfill multiple requirements: it must be biodegradable, non-toxic, and geometrically appropriate, with optimized pore size and morphology to facilitate nutrient exchange and waste removal. Additionally, the scaffold must create a supportive microenvironment that promotes chondrocyte growth and mimics the differential matrix component distribution necessary for cartilage regeneration.

The focus on decellularization in tissue engineering is growing due to its effectiveness in crafting scaffolds that mimic natural tissue characteristics, including minimal immune response, cell compatibility, and decomposability [9]. Wharton's jelly from umbilical cords, abundant in collagen and hyaluronan, exhibits distinctive features that render it a viable option for scaffolds in tissue engineering, especially for cartilage repair by supporting chondrocyte growth [10,11]. In our earlier research, we crafted biological scaffolds from decellularized Wharton's jelly derived from umbilical cords. The scaffolds exhibited a beneficial microstructure, superior mechanical characteristics, and favorable cell compatibility. Additionally, they enhanced cellular chondrogenesis, as shown by the upregulation of collagen II and aggrecan mRNA levels [12]. Based on these findings, we hypothesize that the DUCWJ matrix offers an ideal three-dimensional setting for the attachment and proliferation of allogeneic chondrocytes. The unique features of DUCWJ highlight its potential as a human-derived scaffold source for tissue engineering [13]. Leveraging these advantages, we selected DUCWJ as the material for fabricating an ACI-based scaffold to improve cartilage regeneration outcomes.

Mitochondria, known as the cell's power plants, are crucial in developmental and reparative activities [14,15]. However, mitochondrial dysfunction—characterized by bioenergetic failure, oxidative stress, and mitochondria-mediated apoptosis—has been implicated in various degenerative diseases [16]. Substantial evidence also links mitochondrial impairment to articular injuries and the progression of osteoarthritis (OA) [[17], [18], [19]]. In our previous study, we observed that the post-traumatic articular cartilage microenvironment is marked by significant inflammation. At the same time, chondrocytes that are removed from the in vivo environment and placed into an in vitro 2D environment for culture and expansion are susceptible to mitochondrial damage and lose their mature functional phenotype to fibrochondrocytes. We hypothesize that implanted allogeneic chondrocytes are likely to encounter a similarly hostile environment, characterized by excessive mitochondrial reactive oxygen species (mROS) production, ultimately compromising the efficacy of ACI. This highlights the need for a mitochondrial protectant capable of clearing mROS and restoring mitochondrial function to enhance cartilage repair. Szeto-Schiller-31 (SS31), also known as elamipretide, is a peptide that homes in on mitochondria, binding to cardiolipin and preserving the integrity of the inner mitochondrial membrane as well as the structure of the cristae [[20], [21], [22], [23]]. By doing so, SS31 enhances ATP production and reduces reactive oxygen species [[24], [25], [26]]. Research indicates that SS31, a mitochondria-targeted peptide, can reverse mitochondrial dysfunction and ameliorate conditions in musculoskeletal disease models such as diabetic bone defects, osteoarthritis, and tendinopathy by restoring mitochondrial function and reducing oxidative stress [[27], [28], [29]]. Moreover, clinical trials are underway for SS31, which is showing potential in managing mitochondrial myopathies and Barth syndrome [30,31]. Given SS31's success in addressing mitochondrial dysfunction in these contexts, we hypothesized that it could similarly improve the efficacy of ACI-mediated articular cartilage repair by protecting implanted chondrocytes and supporting their mitochondrial health.

The success of allogeneic chondrocyte implantation (ACI)-mediated articular cartilage repair largely depends on the normal functioning of the implanted chondrocytes. To enhance this process, we developed an SS31-loaded DUCWJ delivery system designed for the sustained release of SS31, aiming to support in situ cartilage regeneration. In this study, we fabricated a decellularized Wharton's jelly (DUCWJ) scaffold using decellularization and freeze-drying techniques. The scaffold was then optimized by incorporating SS31, resulting in the SS31@DUCWJ system. The primary objective was to evaluate the regenerative potential of SS31@DUCWJ as a tissue engineering scaffold to improve ACI-mediated cartilage repair in a rabbit model. This research provides a foundation for developing scaffold-based strategies to enhance the efficacy of ACI-mediated articular cartilage repair and serves as a reference for designing innovative biomaterials for tissue regeneration.

## 2 Methods and material

### 2.1 Isolation and culture of rat articular chondrocytes

Articular chondrocytes from 10- to 12-week-old Sprague–Dawley rats were extracted using established methods, with details provided in the supplementary materials [32]. Briefly, fresh knee articular cartilage was carefully dissected using sterile scissors, with any attached muscle tissue removed. The cartilage was dissected into fragments measuring about 1 mm^3^ and washed with sterile saline to remove any residue and blood. The cartilage fragments underwent three washes with phosphate-buffered saline (PBS), were treated with 0.25 % trypsin for 5 min, and subsequently rinsed with PBS to eliminate any remaining trypsin. Next, the fragments were placed in a serum-free DMEM medium with 0.2 % Type II collagenase and incubated at 37 °C on a shaker for approximately 3–4 h. The digestion was neutralized using DMEM medium with 10 % fetal bovine serum (FBS), and the mixture was passed through a 200 μm single-cell filter to remove undigested fragments, yielding a single-cell suspension. The suspension was centrifuged at 1200 rpm for 10 min at 20 °C. After discarding the supernatant, the cell pellet was resuspended in DMEM medium containing 10 % FBS and transferred to a culture dish. The culture medium was refreshed every three days, and subculturing of cells occurred once they achieved 80–90 % confluence.

### 2.2 Assessment of inflammation, ECM accumulation and apoptosis in chondrocytes

#### 2.2.1 RT-PCR

Rat chondrocytes were seeded into 6-well plates and cocultured under different conditions (P2, P6, and P6+SS31) for 24 h at 37 °C in a 5 % CO_2_ environment. Total RNA was isolated and cleaned from the chondrocytes utilizing the Fast Pure Cell/Tissue Total RNA Isolation Kit V2 (RC112-01, Vazyme, China), following the instructions provided by the manufacturer. Reverse transcription to complementary DNA (cDNA) was performed using the HiScript III All-in-One RT SuperMix Perfect for qRT-PCR (Vazyme, China; R333-01). Quantitative real-time PCR (qRT-PCR) was conducted using a Step-One Real-Time PCR system and Taq Pro Universal SYBR qRT-PCR Master Mix (Vazyme, China; Q712-03). Gene expression levels were standardized against GAPDH, with relative expression determined by applying the 2^−ΔΔCt formula. All experiments were performed in triplicate to ensure reproducibility. The primers used for qRT-PCR are listed in Supplemental Table S1.

#### 2.2.2 Western blotting

Rat chondrocytes were seeded into 6-well plates and cocultured under different conditions (P2, P6, and P6+SS31) for 48 h at 37 °C in a 5 % CO_2_ environment. The cultured chondrocytes were employed for Western blotting to evaluate the levels of proteins involved in inflammation, ECM accumulation and apoptotic signalling. Proteins were extracted, quantified, and loaded onto a 10 % sodium dodecyl sulfate-polyacrylamide gel (SDS-PAGE, PG112, Shanghai Epizyme Biomedical Technology Co., Ltd, China). After electrophoresis, the proteins were transferred onto polyvinylidene fluoride (PVDF) membranes (Millipore, Sigma–Aldrich). The membranes were treated with 5 % skimmed milk for blocking, then incubated with primary antibodies, and subsequently with secondary antibodies. The primary antibodies used included iNOS (Abcam, USA; ab178945), COX-2 (CST, USA; 12282), COL-2 (Abcam, USA; ab34712), ACAN (Affinity Biosciences, USA; AF0135), MMP-13 (Abcam, USA; ab39012), ADAMTS-4 (Abcam, USA; ab185722), BCL-2 (Abcam, USA; ab194583), BAX (CST, USA; 14796), Caspase-3 (CST, USA; 9662) and GAPDH (Abcam, USA; ab181602). After incubation, the membranes were rinsed with TBST and detected using an enhanced chemiluminescence system.

#### 2.2.3 Immunofluorescence assay

Rat chondrocytes were seeded into 24-well plates and cocultured under different conditions (P2, P6, and P6+SS31) for 48 h at 37 °C in a 5 % CO_2_ environment. Post-incubation, the cells were fixed in paraformaldehyde for 20 min, permeabilized with 0.1 % Triton X-100 for 10 min, and blocked with 10 % goat serum for 1 h at 25 °C. The cells were then incubated overnight at room temperature with primary antibodies targeting COX-2 (CST, USA; 12282), MMP-13 (Abcam, USA; ab39012), and COL-2 (Abcam, USA; ab34712). Subsequently, the cells were incubated for 1 h with Alexa Fluor 488-conjugated goat anti-rabbit IgG (ZSGB-BIO, China; ZF-0516). Nuclei were counterstained with DAPI, and the fluorescent images were acquired using a fluorescence microscope (Nikon, Japan; Ni-U).

#### 2.2.4 TUNEL assay

TUNEL was performed on P2 chondrocytes, P6 chondrocytes and SS31-treated P6 chondrocytes, respectively. The apoptosis rate was detected using the TUNEL Apoptosis Detection Kit (Beyotime, China; C1089). PBS rinsed cells were fixed with paraformaldehyde for 30 min and then rinsed again. The cells were incubated with PBS containing 0.3 % Triton X-100 for 5 min at room temperature. Cells were then rinsed with PBS and incubated with the configured Tunel assay for 60 min at 37 °C, protected from light. After a final rinse with PBS, the samples are sealed with an anti-fluorescence quenching sealer and observed under a fluorescence microscope (Nikon, Japan; Ni-U).

### 2.3 Chondrocytes mitochondrial protection evaluation

#### 2.3.1 DCFDA staining

Levels of intracellular reactive oxygen species (ROS) were assessed with the ROS indicator DCFH-DA. Rat chondrocytes were seeded into 6-well plates and co-cultured for 48 h under different conditions (P2, P6 and P6+SS31) at 37 °C in an environment of 5 % CO_2_. Subsequently, the cells were treated with DCFH-DA for 30 min, washed with PBS, and further incubated with Hoechst 33342 (Solarbio, C0031) for 10 min to stain the nuclei. After a final PBS wash, intracellular fluorescence was observed using a fluorescence microscope (Nikon, Japan; Ni-U). The average fluorescence intensity, reflecting ROS levels, was measured using ImageJ software.

#### 2.3.2 JC-1 assay

Mitochondrial membrane potential (Δψm) was evaluated with the JC-1 assay. Rat chondrocytes were seeded into 6-well plates and co-cultured for 48 h under different conditions (P2, P6 and P6+SS31) at 37 °C in an environment of 5 % CO_2_ and subsequently stained with JC-1 for 20 min at 37 °C. Imaging was performed using a confocal laser scanning microscope (TCS-SP5, Leica). In this test, red fluorescence signifies JC-1 aggregates, which denote high mitochondrial membrane potential, whereas green fluorescence indicates JC-1 monomers, which suggest low mitochondrial membrane potential. Mitochondrial membrane potential was determined by measuring the ratio of red to green fluorescence intensities.

### 2.4 Preparation and characterization of SS31@DUCWJ

#### 2.4.1 Preparation of DUCWJ

To prepare decellularized Wharton's jelly (DUCWJ), umbilical cords were first rinsed three times with sterile PBS. The cords were then cut into 3 cm segments, dissected longitudinally, and the amniotic membrane was removed. Following the removal of arteries and veins, the Wharton's jelly was cut into 1–2 mm pellets. The pellets were placed in a solution with 10 mM Tris buffer and 5 mM EDTA and incubated at 4 °C for 10 h. Subsequently, the solution was switched to a mixture comprising pure water, 1 % Triton X-100, 1 M KCl, and 50 mM Tris buffer. The samples were incubated on a shaker at 4 °C for 12 h. The samples were then washed three times with sterile PBS, with each wash duration being 1 h. After washing, the samples were soaked in sterile saline with added RNase (100 μg/mL) and DNase (150 IU/mL), and incubated at 37 °C for 6 h on a shaking platform. Finally, the nuclease-digested samples were washed three additional times with sterile PBS (1 h per wash) and frozen at −80 °C for further use, yielding decellularized Wharton's jelly particles.

#### 2.4.2 Evaluation of DUCWJ

To assess the state of Wharton's jelly pre- and post-decellularization, both the original and decellularized samples were fixed with 4 % paraformaldehyde for 24 h. Samples were embedded in OCT, cut into 8 μm sections, and examined for remaining nuclear debris and ECM components using H&E, Masson's trichrome, and DAPI staining. For microstructural evaluation, primary and decellularised samples were fixed with 4 % paraformaldehyde, freeze-dried, gold-plated and then observed under a scanning electron microscope (SEM). The mechanical properties of both the native (UCWJ) and decellularized (DUCWJ) samples were evaluated using a uniaxial testing device (Optics11 Life, Netherlands; Piuma). Viability of cells on DUCWJ and SS31@DUCWJ scaffolds was determined using a live/dead staining kit (Beyotime, China; C2015M). Cell-scaffold constructs were cultured for 24 h, followed by incubation with calcein-AM and PI in PBS at 37 °C for 30 min. Following PBS washing, the constructs were observed under a fluorescence confocal microscope (Leica, Germany; Leica TCS-SP5), and image analysis was performed using LAS_X software (Leica, Germany). To assess chondrocyte morphology on the DUCWJ and SS31@DUCWJ scaffolds, cell cytoskeleton staining was performed. Cell-scaffold constructs cultured for 24 and 72 h were fixed with 4 % paraformaldehyde and stained with FITC-conjugated phalloidin (Sigma–Aldrich) to visualize F-actin filaments. Nuclei were counterstained with DAPI (Sigma–Aldrich) for 10 min. Fluorescence images were captured using a microscope (Leica, Germany; Leica TCS-SP5).

#### 2.4.3 Preparation of SS31-conjugated DUCWJ (SS31@DUCWJ)

The amide bond was created between the amine groups of the DUCWJ scaffold and the terminal carboxylic groups of SS31 using carbodiimide chemistry, employing a "graft-to" strategy. The reaction was catalyzed by preparing an EDC/NHS solution in 95 % anhydrous ethanol, which included 1-(3-dimethylaminopropyl)-3-ethylcarbodiimide (EDC, 9.6 mg/mL) and N-hydroxysuccinimide (NHS, 2.3 mg/mL) in the correct molar ratio. SS31 was prepared at a 5 mg/mL concentration in the EDC/NHS solution and incubated for 1 h at 25 °C. Subsequently, the DUCWJ scaffold was placed in this solution to interact with the NHS-esterified SS31 for 48 h with gentle stirring. Post-reaction, the SS31-conjugated DUCWJ scaffold (SS31@DUCWJ) was rinsed three times with deionized water and then freeze-dried for future application.

### 2.5 Animal experiments

All procedures involving animals were sanctioned by the Institutional Animal Care and Use Committee at PLA General Hospital. A chondral defect model was established following a previously described protocol [32]. Prior to the experiment, 21 twelve-week-old New Zealand White rabbits (weighing 2.5–3.0 kg) were acclimated to laboratory conditions for one week. Rabbits were assigned randomly across three groups, with three additional rabbits serving as positive controls for sham operations. Each group comprised three rabbits evaluated at two time points: 6 weeks and 12 weeks, with knee surgeries conducted on both sides, resulting in a sample size of *n* = 6 (accounting for both knees of the three rabbits in each group). Under sterile conditions and with anesthesia, a midline incision was made longitudinally to expose the knee joint. In the sham surgery group, the cartilage remained undamaged, and the incisions were closed with sutures. In the other groups, a microdrill was utilized to form a 3.5 mm diameter and 2 mm deep osteochondral defect in the trochlear groove. Saline was used for irrigation during the drilling process to avoid heat-induced damage and keep the tissue moist. Rabbits in the control group received no treatment post-defect creation. In the DUCWJ group, the defects were treated with DUCWJ scaffolds loaded with ex vivo expanded chondrocytes, whereas rabbits in the experimental group were treated with SS31@DUCWJ scaffolds loaded with ex vivo expanded chondrocytes. After implantation, the joint capsule and skin were carefully sutured, and the rabbits were allowed unrestricted movement in their cages post-surgery. At 6- and 12- week post-surgery, the rabbits were euthanized, and the distal femur samples were harvested for further analysis.

### 2.6 Macroscopic evaluation and micro-CT analysis

Macroscopic scoring was conducted independently and blindly by three experienced musculoskeletal researchers, adhering to the ICRS scoring system guidelines (Table S2). At the specified times for harvest, the rabbits were humanely euthanized, and their femurs were retrieved for further analysis. The femoral trochleas were visually inspected, and the findings were documented with photographs. After the macroscopic evaluation, the specimens underwent scanning using the GE Explorer Locus SP system. Three-dimensional (3D) reconstructions were created for each femur, with a region of interest (ROI) being outlined in the regenerated defect area. Within these ROIs, bone volume per tissue volume (BV/TV) and bone mineral density (BMD) were measured to evaluate the progress of cartilage repair.

### 2.7 Histological staining and histological assessment

Knee joints were harvested from rabbits at 6- and 12- week post-surgery. Samples were fixed in 4 % paraformaldehyde (pH 7.4) for 48 h, followed by decalcification in 10 % EDTA for 4 weeks. Post-fixation, samples were shaped, dehydrated, encased in paraffin, and cut into 6 μm sections. Regenerated cartilage was analyzed using hematoxylin and eosin (H&E) staining and Safranin O/fast green (SO/FG) staining. Histological sections were reviewed independently by two experienced reviewers and assessed semiquantitatively using the Mankin scoring system (Table S3) to evaluate the quality of the repaired tissue.

### 2.8 Immunohistochemistry (IHC) staining

After hydration in gradient ethanol from 100 % to 50 %, the sections were incubated in 3 % H2O2 for 15 min. After antigen retrieval by pepsin (Abcam, USA; ab64201) and blocking with goat serum (ZSGB-BIO, China; ZLI-9021), the sections were incubated with primary antibody against COL2 (Abcam, USA; ab307674) overnight at 4 °C. The sections were incubated with secondary antibody and an ultrasensitive DAB kit (Solarbio, China; DA1015) the next day. Images were obtained using a light microscope (Nikon, Japan; Ni-U).

### 2.9 Statistical analysis

Data were analyzed statistically using suitable methods in accordance with the type and distribution of the data. Student's t-test was applied for comparing two groups with parametric data. For parametric data across several groups, one-way ANOVA coupled with Tukey's post hoc test was used, and for comparisons considering various genotypes and treatments, two-way ANOVA with Tukey's test was implemented. The rank-sum test was used to analyze data with nonhomogeneous variance. Normality tests were conducted for all datasets. Statistical processing was conducted utilizing SPSS (version 25.0, Chicago, IL, USA) or GraphPad Prism (version 9.5.1, GraphPad Software Inc., San Diego, CA, USA). Results are presented as means ± standard deviation (SD), and statistical significance was set at p < 0.05.

## 3 Results

### 3.1 SS31 attenuates the inflammatory response that occurs during passaging

**Fig. 1:**
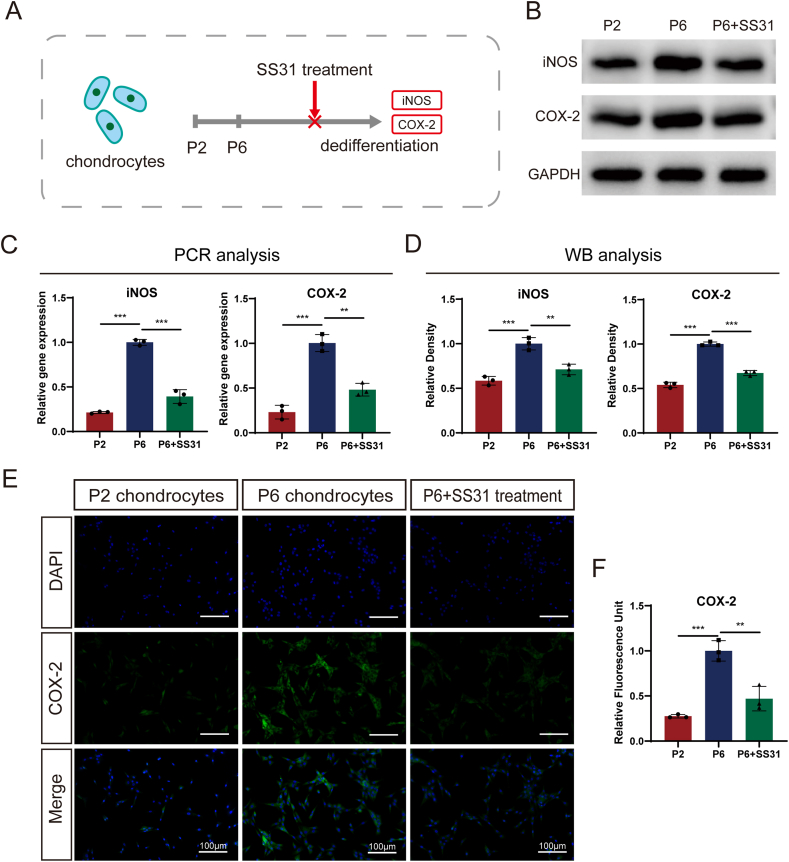
SS31 alleviated the replicative senescence-induced inflammatory response. (A) Schematic graph of this study; (B, C) Levels of iNOS and COX-2 in rat primary chondrocytes as determined by RT-PCR and Western blotting. (D) Quantitative analysis of immunoblotting in (C), assayed by ImageJ program. (E) IF staining of COX-2. Scale bar: 100 μm. (F) Fluorescence intensity analysis, performed using ImageJ program. The values represent the mean ± SD of three independent experiments. ∗p < 0.05 vs. control group.

Reducing inflammation and alleviating the inflammatory response are critical factors in the cartilage defect repair process. To investigate the intensity of the inflammatory response during chondrocyte passaging and the therapeutic effect of SS31, we analyzed mRNA levels 24 h post-treatment and protein levels 48 h post-treatment in rat articular chondrocytes. As shown in Fig. 1A, P2 chondrocytes, P6 chondrocytes, and SS31-treated P6 chondrocytes were included. Results from RT-PCR and immunoblotting analyses (Fig. 1B–C) revealed a significant upregulation of pro-inflammatory molecules, such as iNOS and COX-2, in P6 chondrocytes compared to P2 chondrocytes. However, SS31 treatment markedly reduced the expression of these inflammatory markers. Statistical analysis further confirmed these differences as significant (Fig. 1D). Additionally, immunofluorescence staining (Fig. 1E and F) demonstrated higher cellular expression of COX-2 in the P6 group, which was effectively attenuated following SS31 treatment. These findings highlight the potential of SS31 in mitigating the inflammatory response associated with chondrocyte passaging.

### 3.2 SS31 restored ECM metabolism in multiple passaged chondrocytes

**Fig. 2:**
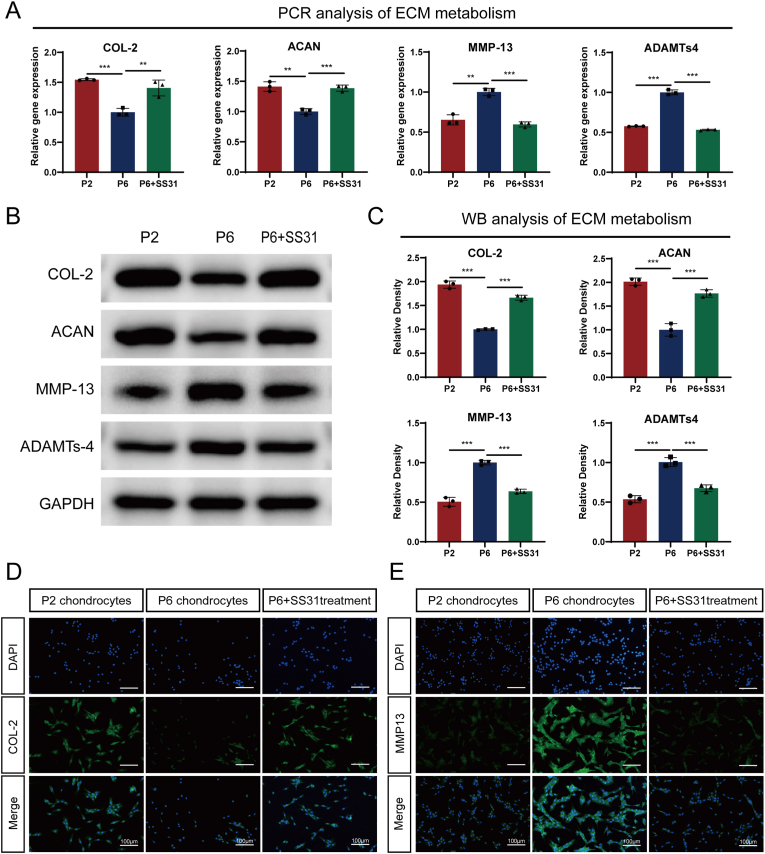
SS31 restrained ECM metabolism. (A) Transcriptional and (B) protein levels of Col-2, Aggrecan, MMP-13 and ADAMTs-4 in cells as determined by RT-PCR and WB method. (C) Quantitative analysis of immunoblotting in (B), assayed by ImageJ program. (D–E) IF staining of COL-2 and MMP13. Scale bar: 100 μm. The values represent the mean ± SD of three independent experiments. ∗p < 0.05 vs. control group.

Articular cartilage is predominantly composed of type II collagen (COL-2) and aggregated proteoglycans (ACAN). The degradation of extracellular matrix (ECM) components is a hallmark of osteoarthritis. Previous studies have demonstrated that chondrocytes undergoing successive passages exhibit reduced matrix secretion and increased matrix degradation. This is characterized by the downregulation of anabolic markers COL-2 and ACAN, and the upregulation of catabolic markers, such as matrix metalloproteinases (MMPs) and ADAMTS. To investigate the role of SS31 in enhancing matrix production in post-passage chondrocytes, we used P2 chondrocytes, P6 chondrocytes and SS31-treated P6 chondrocytes. mRNA expression levels were assessed after 24 h, and protein levels were evaluated at 48 h. As shown in Fig. 2A, successive passages led to decreased mRNA expression of anabolic biomarkers COL-2 and ACAN, alongside increased expression of catabolic biomarkers MMP-13 and ADAMTS-4. Remarkably, treatment with SS31 significantly reversed these trends. Fig. 2B and C demonstrate that SS31 restored protein levels of COL-2 and aggregated proteoglycan while reducing protein levels of MMP-13 and ADAMTS-4 in post-passage chondrocytes. These findings were further corroborated by immunofluorescence staining, as illustrated in Fig. 2D–E and Fig. S1A, highlighting the potential of SS31 to mitigate ECM degradation and promote matrix production in chondrocytes subjected to multiple passages.

### 3.3 SS31 improves oxidative stress and mitochondrial dysfunction in successive chondrocytes

**Fig. 3:**
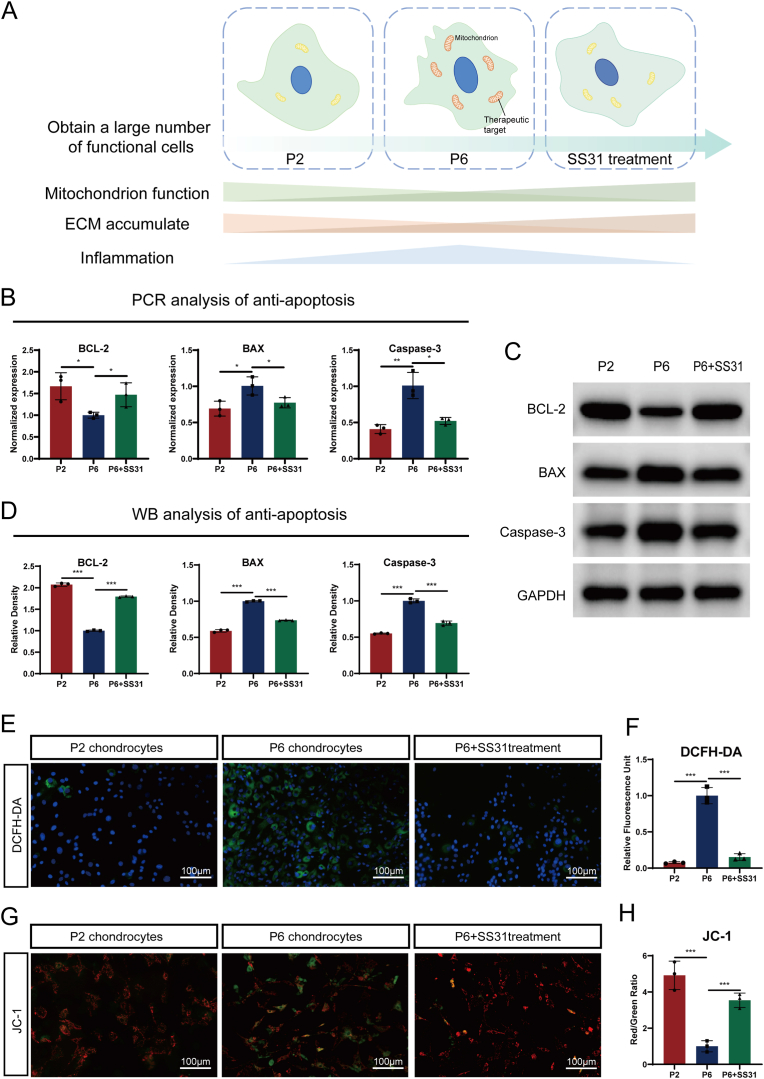
SS31 alleviated the replicative senescence-induced chondrocytes apoptosis. (A) Schematic graph of this study. (B) Transcriptional levels of Bcl-2, Bax and Cleaved Caspase-3 in chondrocytes as determined by RT-PCR. (C)Protein levels of Bcl-2, Bax and Cleaved Caspase-3 in chondrocytes as determined by WB method. (D) Quantitative analysis of immunoblotting in (C), assayed by ImageJ program. (E) DCFDA staining of the chondrocytes of every group. Nuclei were stained with DAPI. Scale bar: 100 μm. (F) Quantitative analysis of mean fluorescence density in every group for DCFH-DA (E) immunostaining. (G) JC-1 assay was applied to detect the mitochondrial membrane potential of chondrocytes in every group. Scale bar,:100 μm. (H) Quantitative analysis of mean fluorescence density in every group for JC-1 (G) immunostaining. The values shown represent the mean ± SD of three independent experiments. ∗p < 0.05 vs. control group.

Prior research has underscored the importance of chondrocyte apoptosis in the regeneration of articular cartilage via autologous chondrocyte implantation (ACI) [1]. In this study, we evaluated the potential of SS31 to alleviate apoptosis in articular chondrocytes by treating P6 chondrocytes with SS31 and comparing them with P2 chondrocytes and P6 chondrocytes (Fig. 3A), and measuring the mRNA and protein levels of apoptosis-related molecules. As shown in Fig. 3B, C and 3D, multiple passages induced significant changes in the transcriptional and translational levels of BAX, Caspase-3, and Bcl-2. Notably, SS31 treatment restored these levels, reversing the effects of passaging. It is well established that in vitro passaging of chondrocytes is often accompanied by dedifferentiation, which leads to increased mitochondrial numbers and, at later stages, structural destruction. We conducted tests using the TUNEL assay to further understand the therapeutic effect of SS31 on P6 chondrocyte apoptosis. The experimental results showed that SS31 significantly reduced the number of more apoptotic chondrocytes in P6 chondrocytes (Fig. S1B). To analyze the impact of successive passaging on mitochondrial function and the protective effects of SS31, we continued our experiments using P2 chondrocytes, P6 chondrocytes and SS31-treated P6 chondrocytes. To assess mitochondrial reactive oxygen species (ROS) levels, a DCFDA staining assay was conducted, as shown in Fig. 3E and F. Additionally, the mitochondrial membrane potential was assessed with JC-1 staining (Fig. 3G and H). The results revealed that successive passaging led to mitochondrial dysfunction, as evidenced by altered ROS levels and disrupted membrane potential. Importantly, treatment with SS31 alleviated these dysfunctions. These findings demonstrate that SS31 effectively counteracts mitochondrial dysfunction caused by successive chondrocyte passaging, thereby supporting normal mitochondrial function and reducing apoptosis.

### 3.4 Characterization of acellular Wharton's jelly

**Fig. 4:**
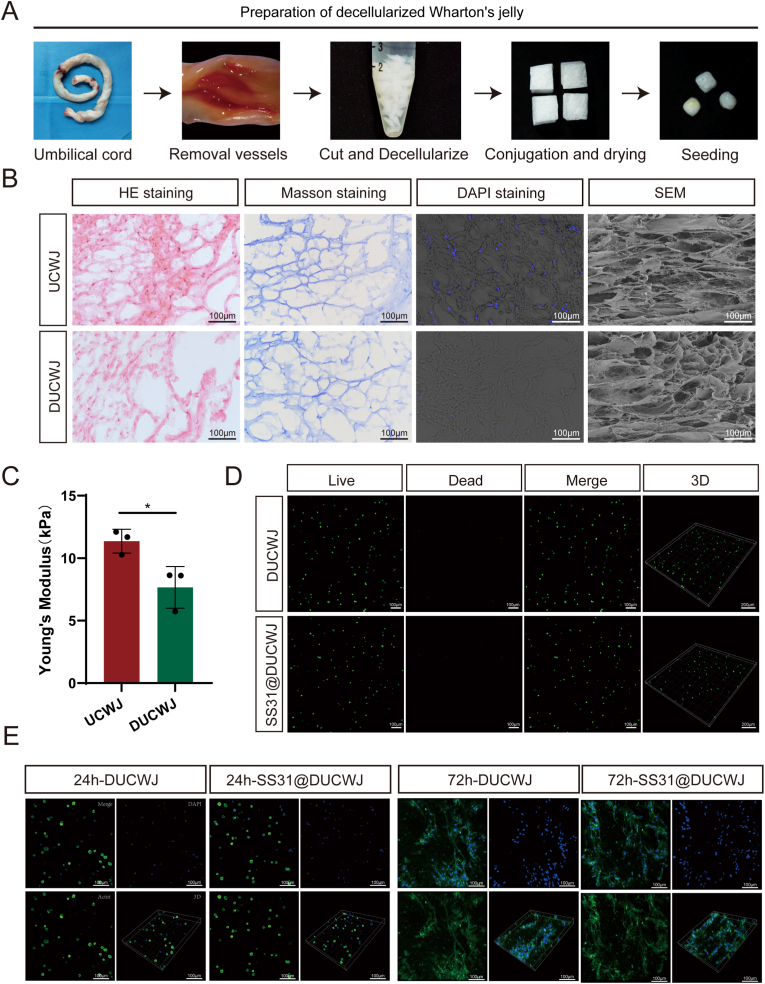
Histological and biocompatibility characterization of the DUCWJ scaffold. **(**A) Schematic graphs of preparation of the DUCWJ scaffold. (B) The histological staining, including HE, Masson's trichrome and DAPI staining, as well as SEM micrographs of the UCWJ before and after decellularization. (C) Young's modulus of the UCWJ before and after decellularization. (D) Live/dead cell analysis for the DUCWJ and DUCWJ@SS31 scaffolds on which rat chondrocytes were seeded for 1 day; representative images show live (green) cells, dead (red) cells, and 3D reconstruction images. (E) The confocal fluorescence images of the rat chondrocytes on the DUCWJ and DUCWJ-SS31 scaffolds for 24 and 72 h (green: cytoskeleton, blue: nuclei); Data are expressed as means ± standard deviation.

As shown in Fig. 4A, maternal umbilical cord tissue was collected, immersed in Dulbecco's phosphate-buffered saline containing 1 % streptomycin and penicillin, and stored at 4 °C. The tissue was then cut into 3 cm segments in a sterile environment, opened longitudinally, and the surface and vascular tissues were removed. Residual blood cells and debris were washed away by repeated rinsing with PBS containing 1 % streptomycin. Subsequently, the tissue was cut into small square pieces measuring 1–2 mm in length. It is important to note that the entire procedure was conducted under sterile conditions. Following decellularization, the DUCWJ pieces appeared as a milky-white, gel-like material. Histological analyses were performed to evaluate the structural integrity of the extracellular matrix (ECM) post-decellularization. Staining with H&E and DAPI (CD31) revealed no detectable nuclear remnants, indicating successful cell removal (Fig. 4B). Furthermore, Masson's staining confirmed that collagen fibers were well-preserved, while scanning electron microscopy (SEM) demonstrated that the decellularized ECM exhibited higher porosity compared to untreated DUCWJ (Fig. 4B).

The mechanical properties of the decellularized ECM were assessed by measuring its Young's modulus, which showed a slight reduction after decellularization(Fig. 4C). Cell viability, assessed by live/dead staining, showed no significant variation between the control and SS31@DUCWJ groups (Fig. 4D). Additionally, as shown in Fig. 4E, cytoskeleton staining indicated that most articular chondrocytes maintained a rounded morphology after 24 h of incubation. By 72 h, cells were evenly spread in both groups, demonstrating consistent compatibility and adaptability.

### 3.5 Cartilage repair by DUCWJ and SS31@DUCWJ

**Fig. 5:**
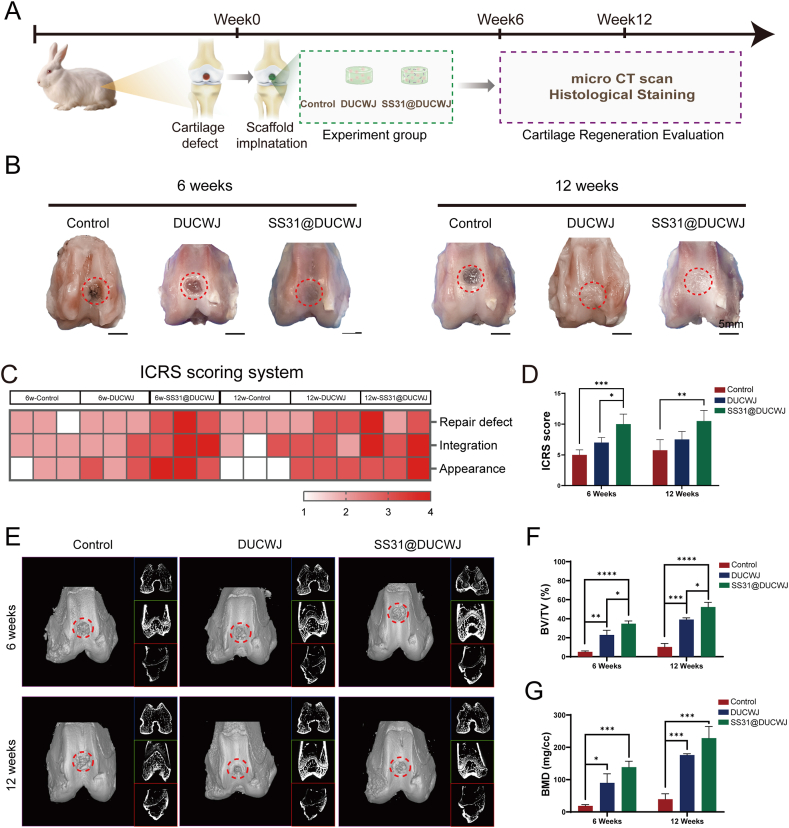
SS31@DUCWJ hydrogels enhance in vivo cartilage regeneration. (A) Schematic illustration of the study design in vivo. (B) Representative macroscopic images of the repaired tissues at 6 and 12 weeks postoperation. Red circles indicate the defect area. (C) Macroscopic scores of repaired cartilage tissue at 6 and 12 weeks according to the ICRS scoring system. (D) ICRS scores of the articular cartilage in each group after 6 weeks and 12 weeks. (E) Micro-CT images showing 2D and 3D reconstruction of the repaired cartilage at 6 and 12 weeks postoperation. Red circles indicate the defect area. (F) Quantitative analysis of BV/TV (n = 6) and G) Tb.N in the defect area (n = 5). Data are means ± SDs (∗p < 0.05, ∗∗p < 0.01, ∗∗∗p < 0.005, ∗∗∗∗p < 0.001, ns. represents no significant difference).

At 6- and 12-week post-surgery, the rabbits were euthanized, and their femoral condyles were harvested for analysis(Fig. 5A). Cartilage repair outcomes were initially evaluated through macroscopic observation. In the control group, defects remained clearly demarcated from surrounding normal tissue at both 6 and 12 weeks (Fig. 5B). In the DUCWJ group, defects were partially covered by a thin layer of fibrous tissue at 6 weeks and fully covered by dense fibrous tissue by 12 weeks (Fig. 5B). In contrast, the SS31@DUCWJ group showed more than 80 % defect coverage by 6 weeks and complete defect repair by 12 weeks, demonstrating superior regenerative outcomes compared to the DUCWJ group. Further evaluation using the ICRS macroscopic scoring system revealed that both the DUCWJ and SS31@DUCWJ treatments significantly improved repair outcomes compared to the control group at both time points (Fig. 5B–D). However, the SS31@DUCWJ group consistently achieved higher scores than the DUCWJ group, highlighting its enhanced effectiveness. Micro-CT imaging supported these findings, showing persistent bone defects in the control and DUCWJ groups, while the SS31@DUCWJ group displayed substantial improvement (Fig. 5E). Measurements of bone volume to tissue volume (BV/TV) and bone mineral density (BMD) quantitatively validated the improved cartilage regeneration in the SS31@DUCWJ group at 6 and 12 weeks (Fig. 5E–G).

**Fig. 6:**
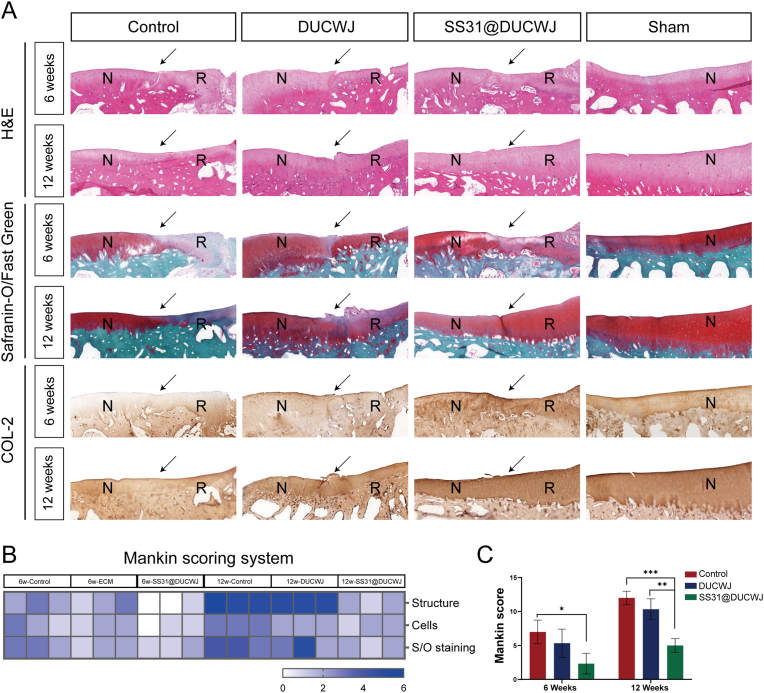
Histological assessments of repaired tissues from the Control, DUCWJ, and DUCWJ@SS31 scaffold groups in vivo. (A) Representative images of staining of repaired cartilage at 6 and 12 weeks for H&E (rows 1 and 2), safranin O/fast green (rows 3 and 4) and COL-II immunohistochemistry (rows 5 and 6). (B) Histomorphology scores of the repaired cartilage tissue at 6 and 12 weeks according to the Mankin scoring system. C) Mankin scores of the articular cartilage in each group after 6 weeks and 12 weeks. The data are shown as the means ± SDs; n = 3; ∗p < 0.05, ∗∗p < 0.01, and ∗∗∗p < 0.005.

Histological analysis with H&E and Safranin O/fast green staining revealed minimal tissue filling and poor cartilage regeneration in the control group. Immunohistochemical staining for COL-II, an important component protein of articular cartilage, also yielded similar results. Both the DUCWJ and SS31@DUCWJ groups demonstrated partial tissue filling and achieved closer tissue thickness to normal cartilage by 12 weeks, though the DUCWJ group exhibited poor integration. In contrast, the SS31@DUCWJ group displayed smoother surface characteristics, thicker tissue, and abundant COL2 deposition, indicative of advanced cartilage regeneration. Scoring using the Mankin system showed significantly better outcomes in the SS31@DUCWJ group, with lower scores reflecting superior repair (Fig. 6A–C). Collectively, these results demonstrate that SS31@DUCWJ hydrogels significantly enhance cartilage regeneration and tissue integration over time, providing superior structural and compositional restoration compared to the control and DUCWJ treatments.

## 4 Discussion

Articular cartilage possesses a restricted capacity for self-repair due to its scant vascularization and the limited regenerative potential of chondrocytes [33]. Cellular therapy has emerged as a pivotal advancement in biotechnology, offering promising therapeutic strategies for cartilage repair [34]. The procedure of autologous chondrocyte implantation (ACI) was initially implemented in 1987 and officially recorded a few years later in 1994 [35]. Initially, ACI involved injecting cultured autologous chondrocytes beneath a flap of autologous periosteal tissue [36]. This technique significantly benefited patients with conditions such as osteochondritis dissecans (OCD) and cartilage defects in the patellofemoral joint, femoral condyle, or tibial plateau. Many experienced symptom reliefs, return to sports, and, in some cases, delayed or avoided the need for joint replacement surgery. To address some limitations of the original technique, a second-generation approach, collagen-covered ACI (CACI), replaced the autologous periosteum with a bioabsorbable collagen membrane [37,38]. This innovation reduced the incidence of graft hypertrophy while delivering clinical outcomes comparable to the original ACI method [38]. Nevertheless, CACI necessitated an open surgical procedure to affix the collagen patch to the lesion. Further advancements led to the development of matrix-induced ACI (MACI), enabling arthroscopic implantation and representing a significant step forward in minimally invasive cartilage repair techniques.

To address the limitations of current approaches, we developed a next-generation three-dimensional MACI system using allogeneic chondrocytes integrated with a novel matrix. Decellularized Wharton's jelly (DUCWJ) from umbilical cords, abundant in hyaluronic acid, collagen, and glycosaminoglycans (GAGs), offers a highly hydrated, tissue-derived scaffold ideal for tissue engineering [13]. Prior research has underscored DUCWJ's suitability as a scaffold material because of its ready availability and distinctive composition. Researchers like Jadalan-Nagari have shown that DUCWJ scaffolds possess biocompatibility, facilitate the adhesion, migration, and expansion of mesenchymal stem cells (MSCs), and promote the regeneration of cartilage and bone [39]. In our own unpublished work, we found that DUCWJ loaded with kartogenin successfully induced tendon-to-bone interface regeneration [40]. This study's histological and biochemical assessments verified that the DUCWJ scaffold is rich in collagen (types I and III) and GAGs, including hyaluronic acid, offering a natural ECM that ensures excellent cell compatibility and stimulates cell growth. Scanning electron microscopy (SEM) and biomechanical tests revealed an optimal three-dimensional porous structure and suitable mechanical strength. In vitro compatibility tests showed that the DUCWJ scaffold supports chondrocyte adhesion and growth, and live/dead assays attested to its lack of cytotoxic effects. One significant challenge with allogeneic chondrocyte implantation is the risk of immune rejection, which can result in the formation of inflammatory fibrous tissue [5]. Effective restoration of chondrocyte function in the hostile microenvironment of injury sites is therefore crucial to improving regenerative outcomes. To address this, we chemically modified the DUCWJ scaffold with mitochondrial protectant SS31 peptides. This modification aimed to regulate mitochondrial function and create an immunomodulatory scaffold system designed to enhance the repair and regeneration of articular cartilage.

Optimizing scaffold performance through peptide modification represents a promising advancement in tissue engineering, offering innovative strategies for system design. SS31, a mitochondria-targeted peptide with antioxidant properties, has gained attention for its ability to enhance cellular uptake and its excellent biosafety profile [41]. In this study, we functionalized the surface of porous DUCWJ scaffolds with SS31 peptides using a carbodiimide chemistry method. This approach leveraged the carboxyl group in SS31, which covalently couples with free amine groups present in the DUCWJ scaffold. Prior research, such as the work by Li et al., demonstrated the potential of mitochondrial-targeting Mn3O4@PDA@Pd-SS31 nanozymes to reduce oxidative stress and reverse mitochondrial dysfunction, yielding promising results for cartilage regeneration and suggesting potential clinical applications in osteoarthritis (OA) treatment [28]. Additionally, mito-protective therapies have been shown to prevent rapid, strain-dependent cartilage damage in OA, emphasizing the importance of targeting mitochondrial dysfunction in cartilage injury treatment [42]. Our findings further support the therapeutic potential of SS31. When applied to senescence-associated inflammatory responses, SS31 significantly inhibited inflammation caused by cell senescence compared to untreated senescent groups, indicating its ability to reduce the inflammatory burden on implanted chondrocytes. Moreover, the aging and loss of chondrocyte-specific characteristics in monolayer cultures typically lead to reduced expression of proteins like collagen type II (Col II) and aggrecan. [43], alongside increased levels of catabolic markers like MMP-13 and ADAMTS-4. In this study, SS31 effectively reversed the senescence-induced loss of Col II and aggrecan, while reducing MMP-13 and ADAMTS-4 levels, highlighting its critical role in mitigating metabolic dysfunction in ACI-mediated cartilage repair.

Previous studies have shown that cellular senescence promotes apoptosis in chondrocytes, primarily through mitochondrial pathways [44]. Aging disturbs the equilibrium between pro-apoptotic Bax and anti-apoptotic Bcl-2, resulting in heightened caspase-3 activity and chondrocyte apoptosis, which exacerbates the degeneration of articular cartilage. In this study, SS31 treatment reduced Bax and cleaved caspase-3 levels while enhancing Bcl-2 expression, suggesting that SS31 mitigates senescence-induced apoptosis in chondrocytes. Mitochondrial impairment is broadly acknowledged as a key element in cartilage damage and restoration [17,45]. Cartilage injury and repair are significantly influenced by mitochondrial dysfunction. Our findings revealed that senescent chondrocytes exhibit elevated reactive oxygen species (ROS) levels and impaired mitochondrial membrane potential compared to healthy controls. This aligns with existing research demonstrating the interplay between ROS and senescence, where excessive ROS production induces oxidative stress, mitochondrial damage, and apoptosis—key contributors to the pathogenesis of cartilage injury and osteoarthritis (OA) [46,47]. Notably, SS31 significantly reversed these ROS-driven effects, reducing oxidative stress, restoring mitochondrial function, and preventing apoptosis in OA chondrocytes [48,49].

To evaluate the in situ regenerative potential of SS31, we established cartilage defect models in New Zealand white rabbits. After sterilization, SS31@DUCWJ scaffolds were implanted into the joint cavities of the experimental group, while unmodified ECM scaffolds were used in the control group. Rabbits treated with SS31@DUCWJ scaffolds demonstrated faster cartilage regeneration and reduced fibrous tissue formation compared to controls. Histological staining further confirmed that SS31@DUCWJ scaffolds promoted better chondrocyte alignment, increased collagen type II expression in regenerated cartilage, and provided superior protection for subchondral bone.

Our findings confirmed that the SS31 peptide exerts significant positive effects on reducing inflammation, regulating extracellular matrix (ECM) metabolism, alleviating chondrocyte apoptosis, and promoting in situ regeneration of damaged articular cartilage (AC) in rabbits. Compared with traditional MACI matrices, such as collagen types I and III, the DUCWJ scaffold developed in this study offers notable advantages, including ease of preparation, enhanced biological functionality, broader therapeutic effects, and reduced side effects. However, there are still challenges and limitations to the application of the SS31@DUCWJ scaffold in AC regeneration. One key issue is the rapid degradation of SS31 in vivo, particularly in the bloodstream, despite its multifunctional protective effects on chondrocytes. To address this, we utilized the DUCWJ scaffold for sustained SS31 release in this study, but further advancements in delivery systems are needed to enhance its stability and efficacy. Additionally, the DUCWJ scaffold, while promising, requires further optimization to address challenges such as standardized preparation methods and effective DNA removal. To maximize the potential of SS31@DUCWJ in promoting ACI-mediated cartilage regeneration, our future work will focus on developing advanced delivery systems and overcoming these limitations to improve its clinical applicability.

## 5 Conclusion

In this study, we developed a peptide-functionalized decellularized DUCWJ scaffold (SS31@DUCWJ) designed to promote cartilage growth and support the normal function of implanted chondrocytes. The scaffold achieved these outcomes by alleviating inflammation, reducing apoptosis, and modulating mitochondrial function in a reactive oxygen species (ROS)-rich microenvironment, ultimately facilitating articular cartilage regeneration. The findings highlight a novel approach to treating articular cartilage defects through modified MACI techniques. This study underscores the critical role of chondrocyte function modulation in managing joint degenerative diseases and advancing cartilage repair strategies.

## Data availability statement

The data that support the findings of this study are available from the corresponding author upon reasonable request.

## Declaration of competing interests

All authors involved in this article declare that there are no conflicts of interest regarding the publication of this paper.
