# Supplementary material for: The mitochondrial protectant SS31 optimized decellularized Wharton's jelly scaffold improves allogeneic chondrocyte implantation-mediated articular cartilage repair
Source: J Orthop Translat. 2025 Apr 15;52:126–37. doi: 10.1016/j.jot.2025.03.023 (PMC12032180; doi:10.1016/j.jot.2025.03.023)
Supplement: Multimedia component 1 [file mmc1.docx]

Supplementary materials for

**The Mitochondrial Protectant SS31 Optimized Decellularized Wharton’s Jelly Scaffold Improves Allogenic Chondrocytes Implantation-Mediated Articular Cartilage Repair**

1. **Materials and Methods**

**1.1 Cell culture**

The isolation and culture of chondrocytes were performed as previously described[1]. In general, cartilage tissue was dissected from rat knees and washed with PBS supplemented with 1% penicillin–streptomycin (Sigma). The cartilage tissue was then cut into small pieces (1 mm3) with ophthalmic scissors and digested with a sterile rotor for 1 h in Dulbecco’s modified Eagle’s medium (DMEM, Corning) containing 0.3% type II collagenase and trypsin. After digestion, the medium was filtered with a 100-μm cell strainer, and the chondrocytes were resuspended in DMEM containing 10% FBS and centrifuged. After centrifugation, the cells were resuspended and transferred to 25 cm^2^ flasks (5% CO_2_, 37 °C). Once the density of chondrocytes reached 80%, they were subcultured with trypsin (Sigma). Cells at the 2nd-6th passages were chosen for the following studies.

1. **Figures and Tables**

**2.1 Figures**


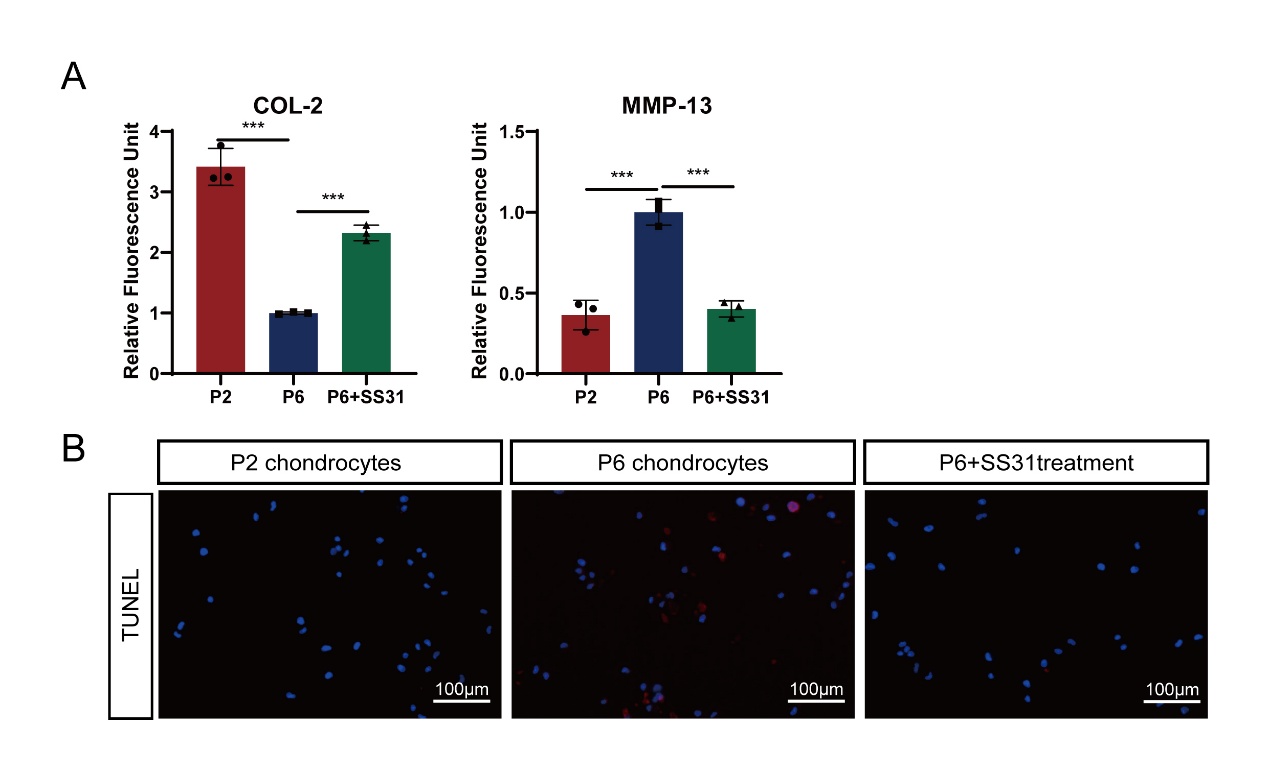


**Figure S1.** (A) Fluorescence intensity analysis of COL-II, MMP-13 was performed using ImageJ program. (B) Tunel staining of chondrocytes. Zoom bar: 100 μm.Values represent the mean ± SD of three independent experiments.* P < 0.05 compared to control.

**2.2 Tables**

**Table S1. Primer sequences used for quantitative real-time PCR.**

| **Primer** | **Forward primers, 5′–3′** | **Reverse primers, 5′–3′** |
| --- | --- | --- |
| Rat - iNOS | GAGACGCACAGGCAGAGGTTG | AGCAGGCACACGCAATGATGG |
| Rat - COX2 | AGGTCATCGGTGGAGAGGTGTATC | CGGCACCAGACCAAAGACTTCC |
| Rat - COL2 | ACGCTCAAGTCGCTGAACAAC | AATCCAGTAGTCTCCGCTCTTCC |
| Rat - aggrecan | GCTACGACGCCATCTGCTACAC | ATGTCCTCTTCACCACCCACTCC |
| Rat - MMP13 | ATACGAGCATCCATCCCGAGACC | AACCGCAGCACTGAGCCTTTTC |
| Rat - ADAMTs4 | GCTTCGCTGAGTAGATTCGTGGAG | TTGACAGGGTTTCGGATGCTTGG |
| Rat - CASP3 | ACGAACGGACCTGTGGACCTG | GTTTCGGCTTTCCAGTCAGACTCC |
| Rat - BCL2 | TACGAGTGGGATACTGGAGATGAAG | TCAGGCTGGAAGGAGAAGATGC |
| Rat - BAX | GGAGACACCTGAGCTGACCTTG | CATCGCCAATTCGCCTGAGAC |
| Rat - GAPDH | ACGGCAAGTTCAACGGCACAG | CGACATACTCAGCACCAGCATCAC |

**Table S2.** International Cartilage Repair Society (ICRS) macroscopic evaluation guidelines.

| ICRS cartilage repair assessment | | Point value |
| --- | --- | --- |
| Degree of defect repair | Level with the surrounding cartilage | 4 |
|  | Repair of 75% of the defect depth | 3 |
|  | Repair of 50% of the defect depth | 2 |
|  | Repair of 25% of the defect depth | 1 |
|  | Repair of 0% of the defect depth | 0 |
| Integration to the border zone | Complete integration with the surrounding  cartilage | 4 |
|  | Demarcating border <1 mm | 3 |
|  | 3/4 of graft integrated with the surrounding cartilage, 1/4 with a notable border >1 mm wide | 2 |
|  | 1/2 of graft integrated with the surrounding cartilage, 1/2 with a notable border >1 mm | 1 |
|  | Ranging from no contact to 1/4 of the graft  integrated with surrounding cartilage | 0 |
| Macroscopic appearance | Intact smooth surface 4 | 4 |
|  | Fibrillated surface 3 | 3 |
|  | Small, scattered fissures or cracks 2 | 2 |
|  | Several small or a few large fissures 1 | 1 |
|  | Total degeneration of the grafted area | 0 |
| Overall repair assessment | Grade I: normal | 12 |
|  | Grade II: nearly normal | 11-8 |
|  | Grade III: abnormal | 7-4 |
|  | Grade IV: severely abnormal | 3-1 |

**Table S3.** Mankin histological evaluation scoring system.

| **I. Structure** | |
| --- | --- |
| Normal | 0 |
| Surface irregularities | 1 |
| Pannus and surface irregularities | 2 |
| Clefts to transitional zone | 3 |
| Clefts to radial zone | 4 |
| Clefts to calcified zone | 5 |
| Complete disorganization | 6 |
| **II. Cells** | |
| Normal | 0 |
| Diffuse hypercellularity | 1 |
| Cloning | 2 |
| Hypocellularity | 3 |
| **III. Safranin O staining** | |
| Normal | 0 |
| Slight reduction | 1 |
| Moderate reduction | 2 |
| Severe reduction | 3 |
| No dye noted | 4 |
| **IV Tidemark integrity** | |
| Intact | 0 |
| Crossed by blood vessels | 1 |
| **Total score** | |
| Minimal | 0 |
| Maximal | 14 |

**Reference**

[1] Z. Tianyuan, D. Haoyuan, L. Jianwei, H. Songlin, L. Xu, L. Hao, Y. Zhen, D. Haotian, L. Peiqi, S. Xiang, A Smart MMP13‐Responsive Injectable Hydrogel with Inflammatory Diagnostic Logic and Multiphase Therapeutic Ability to Orchestrate Cartilage Regeneration, Advanced Functional Materials 33(16) (2023) 2213019.
